# A Patient Presenting with Concurrent Testis Torsion and Epididymal Leiomyoma

**DOI:** 10.1155/2013/485165

**Published:** 2013-05-23

**Authors:** E. Arpali, A. Tok

**Affiliations:** ^1^Department of Organ Transplantation, Istanbul Memorial Hospital, Piyalepaşa Boulevard, Şişli, 34385 İstanbul, Turkey; ^2^Department of Urology, Erzurum Bolge Training and Research Hospital, Dr. Refik Saydam Caddesi Bölge Eğitim ve Araştırma Hastanesi, 25240 Erzurum, Turkey

## Abstract

Leiomyomas are the second most common tumors of epididymis. Patients with leiomyomas are sometimes misdiagnosed with testicular tumors. A Case of a patient with a scrotal mass presenting with testicular torsion is reported. Concurrent occurrence of testicular torsion and epididymal leiomyoma is an extremely rare condition.

## 1. Introduction

Leiomyomas may arise from any tissue that contains smooth muscle. In genitourinary system, their most common locations are mentioned to be uterus and renal capsule [1]. Epididymal leiomyomas are well-defined intrascrotal tumors with a fibrous capsule [1]. Although they are slowly growing tumors which tend to occur in adult age, patients with young age have been reported [1, 2]. These rare benign intrascrotal tumors may lead to orchiectomies because of suspicion of testicular malignity. However, testis torsion due to these paratesticular tumors has never been reported.

## 2. Case Report

**Figure 1:**
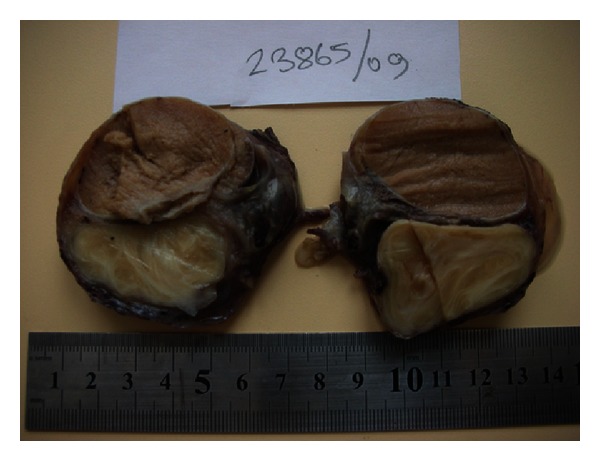
Gross pathological view of the testis and epididymal tumor.

**Figure 2:**
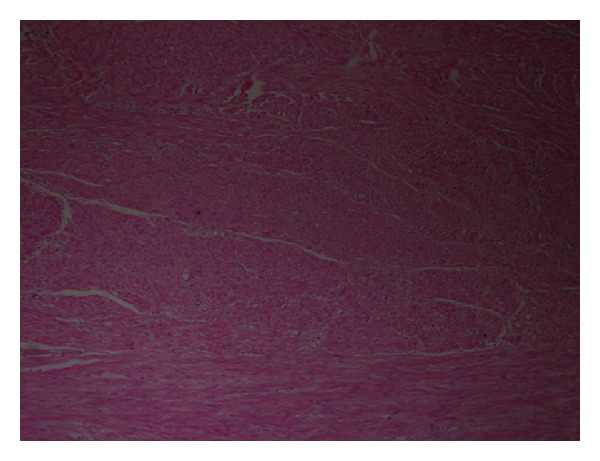
Microscopical view of hematoxylin-eosin stained tumor.

A 42 year-old man presented to emergency department because of sudden left scrotal pain and a scrotal mass. He stated that he had had the mass for one year but the pain had started 4 hours ago and gradually increased. He defined the pain as sharp and continuous in nature which radiated to left inguinal region. Past medical history of the patient was unremarkable. Physical examination revealed a tender, erythematous right scrotum with a nontransilluminating mass of 4 cm which was contiguous to testis. Routine laboratory tests, including total blood count, blood biochemistry, and urinalysis showed no pathologic results. Testicular tumor markers (alfa-fetoprotein, beta human chorionic gonadotropin) were also evaluated owing to the palpated testicular mass. However, the results were within normal limits. Scrotal doppler ultrasonography assessment indicated findings consistent with a solid scrotal mass and concurrent testis torsion but the origin of the mass, whether testis or epididymis, could not be delineated. The patient was informed and prepared for an operation with the prediagnosis of testicular torsion and testis tumor. An inguinal approach was preferred for observation of testis and paratesticular structures. On gross examination during operation the mass was observed to be fixed to both testis and epididymis and radical orchiectomy was performed. Gross pathological examination revealed a white mass in which a whorled pattern could be easily observed on the cut surface (Figure 1). Microscopic evaluation of the material showed interlacing uniform spindle cells without cellular atypia or mitosis (Figure 2). The materials were also desmin and myoglobin positive on immunohistochemical evaluation. No postoperative early surgical complication occurred during hospital stay. There was no evidence of any recurrence of the disease during the first 12 months of follow-up.

## 3. Discussion

Not many cases exist in the literature reporting the testicular masses presenting with testicular torsion. And most of the cases reported are the malignant tumors of the testis [3–7].

To our knowledge no cases of testicular torsion with epididymal leiomyoma has been reported. Tumors of epididymis are rare cases. Following adenomatoid tumors in incidence, leiomyomas have been reported to be the second most common tumor of epididymis [5]. Its incidence has been mentioned to be between 6 and 40% of all epididymal tumors in previous series. It may be encountered in every age group but fifth decade is stated to be the most common period of diagnosis [1, 8, 9].

Macroscopically leiomyomas are tumors which are encircled with a gray-white capsule. A whorled pattern is usually observed on the cut surfaces. Microscopically, interlacing smooth muscle fibers on a fibrous and sometimes hyalinized stroma are characteristic features of the tumor [2]. Leiomyomas are benign tumors; their recurrence after surgery has not been reported in the literature [1].

Physical exam and sonographic evaluation can usually define paratesticular lesions. However it is not dependable enough in distinguishing malign paratesticular lesions from benign ones [9].

 Most of the lesions can be dissected easily from the testis but in our case the lesion could not be dissected from the testis and the origin of the mass could not be defined precisely. Because the malignancy risk could not be eliminated, radical orchiectomy was performed. If epididymal origin is considered during surgery, frozen section can be used to confirm the benign nature of the lesion and testis-sparing surgery can be performed for the preservation of the fertility.

Tendency to testis torsion due to paratesticular mass has not been established. A thorough evaluation in every patient admitted for any scrotal disease should be performed.
